# Diversity of *Saccharomyces cerevisiae* Strains Isolated from Two Italian Wine-Producing Regions

**DOI:** 10.3389/fmicb.2016.01018

**Published:** 2016-06-30

**Authors:** Angela Capece, Lisa Granchi, Simona Guerrini, Silvia Mangani, Rossana Romaniello, Massimo Vincenzini, Patrizia Romano

**Affiliations:** ^1^School of Agricultural, Forestry, Food and Environmental Sciences, University of Basilicata, PotenzaItaly; ^2^Department of Management of Agricultural, Food and Forestry Systems, University of Florence, FlorenceItaly

**Keywords:** *Saccharomyces cerevisiae*, wine, *terroir*, *Aglianico del Vulture*, *Sangiovese*, yeast diversity, genotyping, fermentation products

## Abstract

Numerous studies, based on different molecular techniques analyzing DNA polymorphism, have provided evidence that indigenous *Saccharomyces cerevisiae* populations display biogeographic patterns. Since the differentiated populations of *S. cerevisiae* seem to be responsible for the regional identity of wine, the aim of this work was to assess a possible relationship between the diversity and the geographical origin of indigenous *S. cerevisiae* isolates from two different Italian wine-producing regions (Tuscany and Basilicata). For this purpose, sixty-three isolates from *Aglianico del Vulture* grape must (main *cultivar* in the Basilicata region) and from *Sangiovese* grape must (main *cultivar* in the Tuscany region) were characterized genotypically, by mitochondrial DNA restriction analysis and MSP-PCR by using (GTG)_5_ primers, and phenotypically, by determining technological properties and metabolic compounds of oenological interest after alcoholic fermentation. All the *S*. *cerevisiae* isolates from each region were inoculated both in must obtained from *Aglianico* grape and in must obtained from *Sangiovese* grape to carry out fermentations at laboratory-scale. Numerical analysis of DNA patterns resulting from both molecular methods and principal component analysis of phenotypic data demonstrated a high diversity among the *S*. *cerevisiae* strains. Moreover, a correlation between genotypic and phenotypic groups and geographical origin of the strains was found, supporting the concept that there can be a microbial aspect to *terroir*. Therefore, exploring the diversity of indigenous *S*. *cerevisiae* strains can allow developing tailored strategies to select wine yeast strains better adapted to each viticultural area.

## Introduction

Traditionally, *Saccharomyces cerevisiae* is the predominant yeast species in spontaneous wine fermentations and thus it is the main responsible for the chemical and sensory properties of wines (Pretorius, 2000; Fleet, 2003; Romano et al., 2003; Cocolin et al., 2004; Camarasa et al., 2011). During the last decades, a large number of surveys, based on different molecular techniques analyzing DNA polymorphism, have demonstrated that this species is characterized by a high genetic diversity (Frezier and Dubourdieu, 1992; Querol et al., 1994; Guillamón et al., 1996; Sabate et al., 1998; Pramateftaki et al., 2000; Torija et al., 2001; Schuller et al., 2005; Agnolucci et al., 2007; Romano et al., 2008; Sun et al., 2009; Csoma et al., 2010; Mercado et al., 2011; Capece et al., 2013). In spite of the occurrence of a high number of different *S. cerevisiae* strains at the beginning of the fermentation, it was pointed out that, usually, only few strains (from one to three) dominate the process in the latter stages. Some *S. cerevisiae* strains were isolated over several years in the same cellar as predominant microbiota in wine fermentations (Sabate et al., 1998; Gutièrrez et al., 1999; Augruso et al., 2006) so that the existence of a “winery effect” was suggested (Vezinhet et al., 1992). Alternatively, specific *S. cerevisiae* strains were widespread in different cellars of the same wine-producing region (Versavaud et al., 1995; Blanco et al., 2006) and they were considered representative of an oenological area (Guillamón et al., 1996; Torija et al., 2001). More recently, biogeographical characterization of *S. cerevisiae* wine yeasts carried out at scales above 100 km, has revealed the presence of regional population with specific genotype but no differentiation within the region (Knight and Goddard, 2015). These findings suggest that specific native strains could be associated with a *terroir*, a term that classically includes only grape variety, climate and soil as fundamental factors determining the typical nature of wines (Van Leeuwen and Seguin, 2006) and that might be revised including also a “microbial aspect” (Bokulich et al., 2014; Taylor et al., 2014). Since it is well established that chemical and sensory properties of some wines reflect their geographic origin (Villanova and Sieiro, 2006; Callejon et al., 2010) it was interesting to determine whether regionally defined *S. cerevisiae* genotypes actually exhibit specific metabolic profiles (or phenotypes) able to modulate the wine quality, thus contributing to *terroir*-associated wine characteristics. Indeed, in a recent study Knight et al. (2015) demonstrated significant correlation between the region of isolation of *S. cerevisiae* and aroma profile in wines. The evidence that certain regions have “signature” *S. cerevisiae* populations that can produce significantly different chemical and sensory profiles of wine is of relevance to the wine industry because it may link territory, environment, and final products for wine valorisation (Torija et al., 2001; Romano et al., 2003; Aa et al., 2006; Camarasa et al., 2011; Pretorius et al., 2012; Tofalo et al., 2013). For this reason, the demand of indigenous *S. cerevisiae*, which could be representative of a specific oenological area, is increasing (Orlić et al., 2010). In fact, each strain of *S. cerevisiae* is able to produce different types and quantities of secondary compounds, which are determinant on the desirable organoleptic characteristics of a wine (Pretorius, 2000; Romano et al., 2003; Barrajón et al., 2011; Scacco et al., 2012). Since to perform a better control of the alcoholic fermentation in the modern winemaking the use of yeast starter cultures is diffused, selecting the proper yeast strain can be critical for the development of the desired wine style. Moreover, by using these selected yeast starter cultures, that are better adapted to the environmental conditions, the must fermentation can occur in the correct way (Callejon et al., 2010). In this perspective, the goal of this study was to investigate a possible relationship between the diversity and the geographical origin of indigenous *S. cerevisiae* isolated from two different Italian wine-producing regions (Tuscany and Basilicata) considering two regional grape varieties usually used to produce Controlled Designation of Origin (DOC) wines. Such studies are of great interest in order to establish the existence of typical *S. cerevisiae* strains that would then be useful as inocula in the vinifications carried out in the specific oenological areas (Gutièrrez et al., 1999). The use of autochthonous yeast strains, besides assuring the maintenance of the typical sensory properties of the wines of any given region, can contribute to promote or retain the natural *S. cerevisiae* biodiversity.

## Materials and Methods

### Yeast Strains

Sixty-three *Saccharomyces cerevisiae* isolates were used. The yeasts were previously isolated from spontaneously fermented grape musts of two varieties: “Aglianico del Vulture,” Basilicata region (coded with R1-R33) and “Sangiovese,” Tuscany region (coded with R34-R63). The isolates were maintained on YPD medium [1% (w/v) yeast extract, 2% (w/v) peptone, 2% (w/v) glucose, 2% (w/v) agar].

### Genotypic Characterization of *S. cerevisiae* Isolates

Differentiation between the 63 indigenous *S. cerevisiae* isolates was performed by two molecular methods: microsatellite-primed PCR (MSP-PCR) by using the synthetic oligonucleotide (GTG)_5_ (Orlić et al., 2010) and mitochondrial DNA restriction analysis (mtDNA-RFLP) by using the restriction endonucleases *Rsa*I according to Granchi et al. (2003). Genomic DNA was extracted using a synthetic resin (Instagene Bio-Rad Matrix), following the protocol described in Capece et al. (2011). Amplification reactions were performed in a final volume of 50 μL containing 10 μL 5X Buffer (Promega), 4.0 μL of 25 mM MgCl_2_ (Promega), 1 μL of 10mM dNTP (Promega), 5 μL of 5 μM primer, 0.25 μL (5 U/μL) of *Taq* DNA polymerase (Promega) and 5 μL of the extracted DNA, by adding sterile water until final volume. The thermal cycler was programmed as follows: initial denaturation at 95°C for 5 min, 35 cycles at 94°C for 1 min for denaturing, 1 min at 52°C, 2 min at 72°C for extension and a final step at 72°C for 5 min. PCR products were analyzed by electrophoresis in 1.2% (w/v) agarose gel. The obtained profiles were submitted to cluster analysis using “Complete Linkage” method with Pearson distance by FPQuest software v.4.5 (Bio-Rad).

DNA digestions were performed with the enzyme *Rsa*I and restriction DNA fragments were separated on 0.8% (w/v) agarose gels containing ethidium bromide (1 μg mL^-1^) by electrophoresis in 1X⋅TBE buffer (90 mM Tris-borate, 2 mM, EDTA pH 8.0) at 4 V cm^-1^ for 6 h. The obtained patterns were submitted to pairwise comparison with the Dice coefficient (SD) (Sneat and Sokal, 1973) and cluster analysis with unweighted pair group method (UPGMA) by GelCompar 4.0 software (Applied Math, Kortrijk, Belgium).

### Technological Characterization of *S. cerevisiae* Isolates

The 63 isolates were submitted to screening for some phenotypic properties, such as sulfur dioxide, ethanol and copper resistance and fermentative performance. The SO_2_ and ethanol resistance was evaluated on agarized grape must (pH 3.6), added with increasing doses of K_2_S_2_O_5_ (100–300 mg L^-1^) and ethanol (10–18% vol/vol), respectively. Copper resistance was evaluated on agarized synthetic medium, containing 6.7 g L^-1^ YNB (Yeast Nitrogen Base without amino acids and sulfate), 20 g L^-1^ glucose, added with increasing doses of CuSO_4_ (50, 100, 200, 300, 400, and 500 μmolL^-1^). The strain resistance to the three compounds was evaluated on the basis of positive growth after incubation at 26°C for 24 h, in comparison to the control (the medium without the compound). The degree of resistance of each strain was reported as minimal dose of compounds allowing the growth. All the tests were carried out in duplicate.

### Laboratory-Scale Fermentations

The fermentative performance of the 63 *S. cerevisiae* isolates was tested in inoculated fermentations in two different grape musts, “Aglianico del Vulture” and “Sangiovese” possessing, respectively, the following physico-chemicals characteristics: pH: 3.7 and 3.2; sugars (g L^-1^): 227 and 214; yeast assimilable nitrogen (mgL^-1^): (130 ± 1.4) and (120 ± 2.5). The fermentations were performed according to Capece et al. (2012): 130-mL Erlenmeyer flasks were filled with 100 mL of the two grape musts and added with 50 mg L^-1^ of SO_2_. Each strain was inoculated in grape must at a concentration of 10^6^ cells mL^-1^, from a pre-culture grown for 48 h in the same must. The fermentation was performed at 26°C and the fermentative course was monitored by measuring weight loss, determined by carbon dioxide evolution during the process. At the end of the process, indicated by constant weight of the samples, the wine samples were refrigerated at 4°C to clarify the wine, racked and stored at -20°C until required for analysis. All the experiments were performed in duplicate. Fermentation vigor was measured as weight loss after 2 days of incubation at 26°C, whereas the fermentative power was defined as total weight loss, detected at the end of the process.

### Chemical Analysis

In grape musts, α-amino acid and ammonium concentrations were determined by the NOPA procedure (Dukes and Butzke, 1998) and enzymatic assay according to the manufacturer’s instructions (STEROGLASS s.r.l., Perugia), respectively. Glucose, fructose, ethanol, glycerol, 2,3-butanediol and acetic acid concentrations in experimental wines were determined by HPLC, according to Schneider et al. (1987) and Granchi et al. (1998), utilizing a MetaCarb H Plus Column (8 μm particle, 300 × 7.8 mm; Varian Inc.) and a Pro-star 210 chromatograph equipped with a Refractive Index Detector, in series (Varian Inc.). Higher alcohols (1-propanol, isobutanol, *n*-butanol, 2-methyl-1-butanol, 3-methyl-1-butanol), acetoin, diacetyl, acetaldehyde and ethyl acetate were analyzed by gas chromatography equipped with glass column (6.6% CW 20M BA 80/120 225, 2 m × 6 × 2 mm) as described by Romano et al. (1999).

### Statistical Analysis

The raw data obtained by HPLC and GC analysis were subjected to Principal Component Analysis (PCA) and *t*-test by Statistica software (version 7, StatSoft, Tulsa, OK, USA).

## Results

### Genotypic Characterization of *S. cerevisiae* Isolates

**FIGURE 1:**
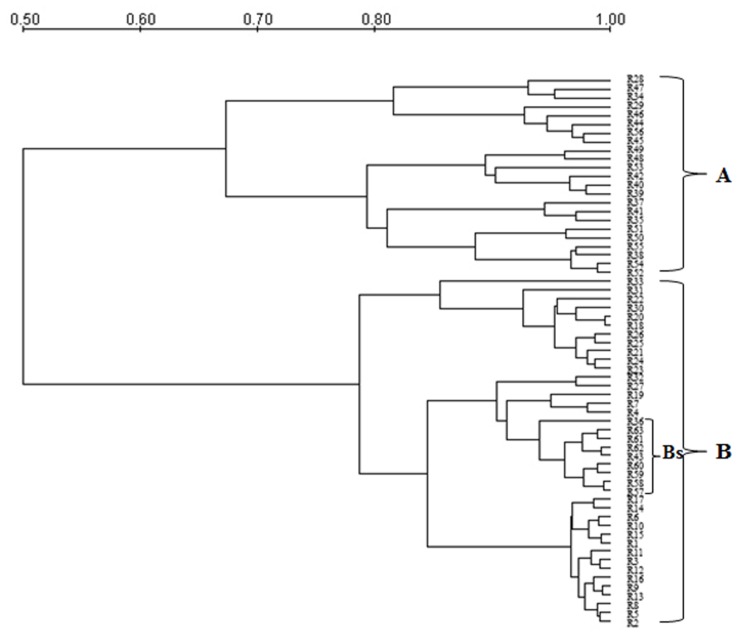
**Dendrogram from UPGMA clustering analysis, based on Pearson coefficient, of the profiles obtained by MSP-PCR of the *S. cerevisiae* isolates from the Aglianico del Vulture (cluster B) and Sangiovese (cluster A) grape must.** Bs indicates a sub-cluster including 9 *S. cerevisiae* isolates from Sangiovese.

Genetic polymorphism in *S. cerevisiae* isolates from Aglianico del Vulture and Sangiovese grape musts was evaluated by MSP-PCR analysis with the (GTG)_5_ primer and mt-DNA-RFLP. MSP-PCR profiles contained a variable number of bands, some of which were common to numerous isolates. The dendrogram resulting from MSP-PCR analysis (**Figure 1**), by considering a similarity coefficient of about 65%, revealed the presence of two clusters (A and B). For the majority of isolates, the distribution was related to yeast origin; in fact, the cluster A grouped isolates from Sangiovese grape must (except R28 and R29 strains), whereas the cluster B grouped all the isolates from Aglianico del Vulture grape must, except the Bs sub-cluster, which contains 9 *S. cerevisiae* isolates from Sangiovese grape must.

**FIGURE 2:**
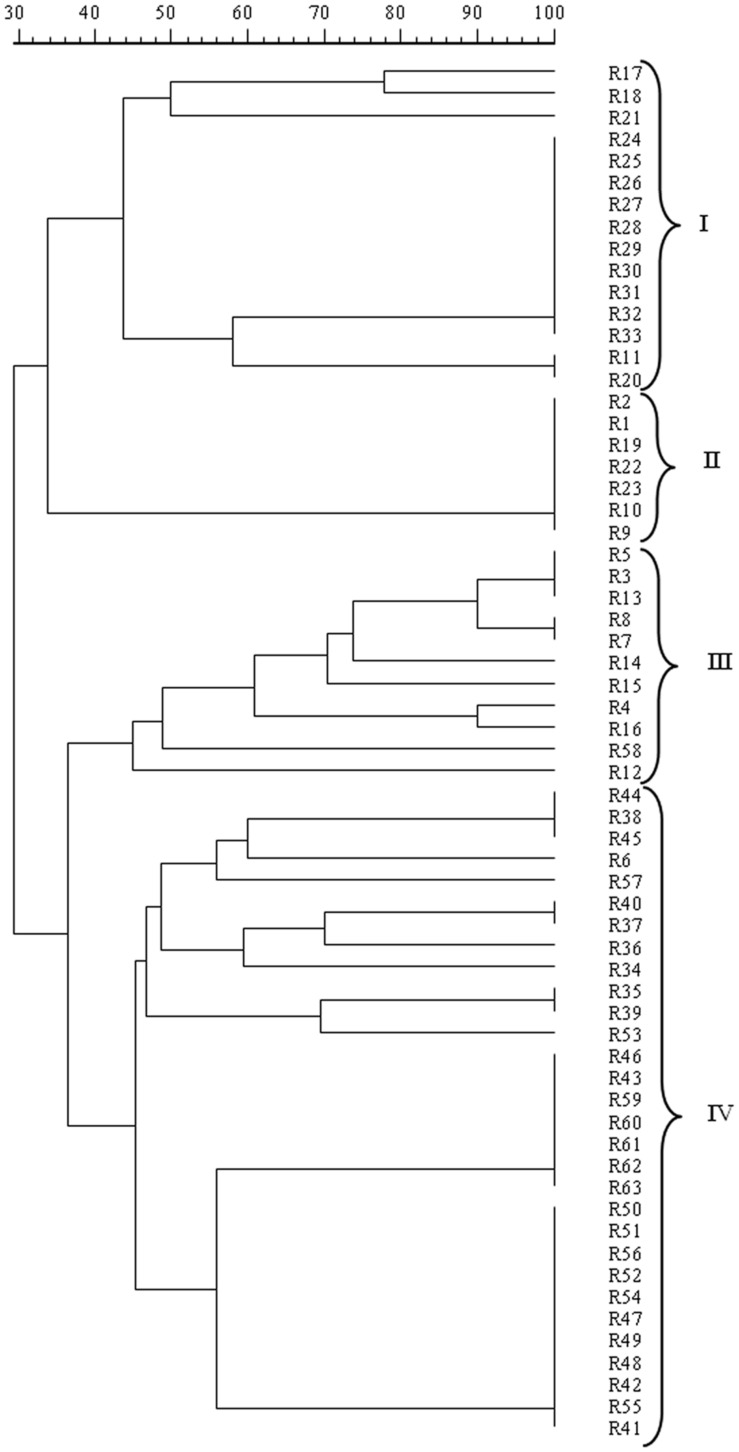
**Dendrogram from UPGMA clustering analysis, based on Dice coefficient of mtDNA *Rsa*I restriction patterns of the *S. cerevisiae* isolates from Aglianico del Vulture (clusters I, II, and III) and Sangiovese (cluster IV) grape must**.

The mitochondrial DNA-RFLP analysis revealed the presence of 24 different patterns, i.e., 24 strains, among the 63 isolates analyzed, confirming the high polymorphism found in *S. cerevisiae* populations. The resulting dendrogram from UPGMA analysis of the patterns obtained with *Rsa*I, reported in **Figure 2**, indicated that *S. cerevisiae* isolates at 40% similarity grouped into four clusters, I, II, III, and IV. All the isolates from Aglianico del Vulture grape must, except for R6, were included in the clusters I, II, and III while all the isolates from Sangiovese grape must, except for R58, were comprised in the cluster IV. Therefore, the analysis pointed out a possible grouping of the assayed *S. cerevisiae* isolates according to their geographic origin.

### Technological Characterization of *S. cerevisiae* Isolates

**FIGURE 3:**
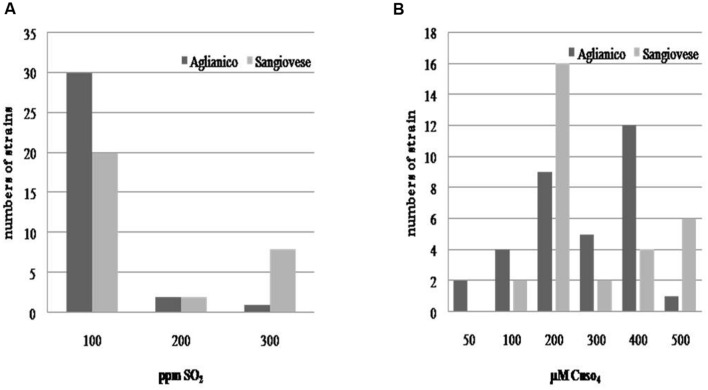
**Distribution of *S. cerevisiae* according to their resistance to sulfur dioxide **(A)** and copper (B)**.

As regards the evaluation of technological characters, the isolates exhibited a high tolerance to ethanol; almost all isolates tolerated 16% v/v of this compound (only few strains developed until 18% v/v of ethanol), whereas a certain variability was found for sulfur dioxide (**Figure 3A**) and copper (**Figure 3B**) resistance. Although the most of isolates exhibited a low tolerance to sulfur dioxide (100 mg L^-1^of SO_2_), all the yeasts developing to the highest tested doses of SO_2_ were isolated from “Sangiovese” (except one). As regards copper tolerance, the isolates were distributed in different classes of resistance. A high number of Sangiovese isolates tolerated 200 μM CuSO_4_, whereas the majority of “Aglianico” isolates developed between 200 and 400 μM CuSO_4_. However, also for copper tolerance, the yeasts growing at the highest copper doses (500 μM CuSO_4_) were mainly Sangiovese isolates.

### Inoculated Fermentation at Laboratory-Scale

**FIGURE 4:**
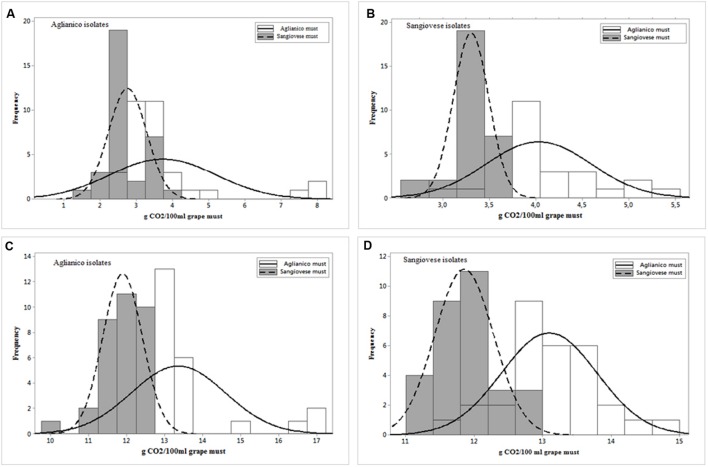
**Distribution of *S. cerevisiae* isolated from Aglianico del Vulture and Sangiovese according to fermentative vigor (g CO_2_/100 mL after 2 days of fermentation) (**A,B**, respectively) and according to fermentative power (g CO_2_/100 mL at the end of fermentation) (**C,D**, respectively) in Aglianico del Vulture and Sangiovese grape must**.

The wild strains were tested in inoculated fermentations at laboratory scale. Two different red musts, “Aglianico” (the isolation grape must of the R1-R33 isolates) and “Sangiovese” (the isolation grape must of the R34-R63 isolates), were used to evaluate strain fermentative performance. The data related to fermentative vigor of Aglianico isolates (**Figure 4A**) indicate that these isolates have shown a different vigor in the two musts; in particular, the isolates exhibited a lower vigor in Sangiovese must (mean value 2.76 g CO_2_/100 mL) than in Aglianico (mean value 3.74 g CO_2_/100 mL). Furthermore, these isolates showed higher variability in Aglianico than in Sangiovese must and the highest fermentative vigor was found in Aglianico isolates fermenting the same isolation grape must (8.24 g CO_2_/100 mL, **Figure 4A**). Also Sangiovese isolates (**Figure 4B**) showed different fermentative vigor in the two grape musts. The results obtained for Sangiovese isolates were similar to data found in Aglianico yeasts, with highest vigor and variability in Aglianico fermentation, although the maximum fermentative vigor of Sangiovese isolates (5.48 g CO_2_/100 mL, **Figure 4B**) was lower than maximum value showed by Aglianico isolates.

The data related to fermentative power, showed in **Figures 4C,D**, confirmed the results obtained for fermentative vigor, with different fermentative behavior in the two musts. As already found for fermentative vigor, Aglianico isolates (**Figure 4C**) showed higher fermentative power than Sangiovese isolates (**Figure 4D**), and both isolates groups showed the best performance in Aglianico grape must, with the highest value of 17.23 g CO_2_/100mL for Aglianico isolates (**Figure 4C**) and 14.8 g CO2/100 mL for Sangiovese isolates (**Figure 4D**). Furthermore, the isolates exhibited higher variability for this parameter in Aglianico than in Sangiovese must (values ranging between 11.76–17.23 and 9.96–12.7 g CO_2_/100 mL, respectively). Finally, the best value was exhibited in Aglianico must by strains isolated from this variety.

The content of 14 yeast metabolic compounds (ethanol, glycerol, acetic acid, acetaldehyde, 1-propanol, 3-methyl-isobutanol, *n*-butanol, 2-methyl-1-butanol, 3-methyl-1-butanol, diacetyl, acetoin, meso and racemic 2,3-butanediol, ethyl acetate) was determined in the experimental wines obtained at the end of the alcoholic fermentations of Aglianico and Sangiovese grape musts.

Statistical analysis of metabolites produced (**Table 1**) demonstrated that, independently of the grape must variety fermented (Aglianico or Sangiovese), some compounds showed significant differences which may be related to the different origin of the yeast strains carrying out the fermentative process. In particular, *S. cerevisiae* isolates from Aglianico produced significantly higher amounts of acetic acid, acetaldehyde, acetoin, 1-butanol and 2,3-butanediols, while *S. cerevisiae* isolates from Sangiovese yielded higher concentrations of 2-methyl-1-butanol and 3-methyl-1-butanol (**Table 1**). The metabolic profiles obtained confirm the wide phenotypic variability within *S. cerevisiae* species, but, in any case, wine composition, independently of the grape variety, was markedly characterized by metabolites of the fermenting yeast strain.

**Table 1:** Statistical analysis (*t*-test *p* < 0.05) of metabolites from fermentations of Aglianico del Vulture grape must (Ag fermented must) and Sangiovese grape must (Sg fermented must) carried out by *S. cerevisiae* isolates from Aglianico del Vulture (Ag) and Sangiovese (Sg), (Values as means ± SD) (different letters indicate significant differences among metabolites produced in the same must).

Compounds		Ag fermented must		Sg fermented must	
		Ag isolates	Sg isolates	Ag isolates	Sg isolates
1-propanol	mgL^-1^	50.57 ± 15.40	48.27 ± 11.67	37.62^b^ ± 11.09	44.00^a^ ± 7.94
Isobutanol	mgL^-1^	100.27^b^ ± 24.25	115.38^a^ ± 33.25	95.80 ± 28.75	91.54 ± 22.00
n-butanol	mgL^-1^	6.17^b^ ± 3.00	3.28^a^ ± 1.25	3.25^b^ ± 2.10	1.59^a^ ± 0.54
2-methyl-1-butanol	mgL^-1^	33.56^b^ ± 9.78	40.33^a^ ± 9.86	27.78^b^ ± 7.01	36.86^a^ ± 7.21
3-methyl-1-butanol	mgL^-1^	275.19^b^ ± 81.82	316.71^a^ ± 74.89	227.33^b^ ± 64.35	286.67^a^ ± 34.47
Acetaldehyde	mgL^-1^	105.31^b^ ± 39.75	79.28^a^ ± 20.57	173.27^b^ ± 87.16	102.32^a^ ± 41.42
Acetoin	mgL^-1^	42.78^b^ ± 24.78	27.17^a^ ± 5.46	59.36^b^ ± 40.80	17.44^a^ ± 7.59
Diacetyl	mgL^-1^	3.51^b^ ± 2.51	5.14^a^ ± 1.09	6.10^b^ ± 2.60	2.50^a^ ± 1.94
Ethyl acetate	mgL^-1^	15.73^b^ ± 10.19	24.70^a^ ± 6.93	34.13^b^ ± 14.93	18.90^a^ ± 5.33
Glycerol	gL^-1^	7.18 ± 0.74	7.39 ± 0.46	6.24^b^ ± 1.51	7.67^a^ ± 0.48
Acetic acid	gL^-1^	0.37^b^ ± 0.11	0.28^a^ ± 0.10	0.52^b^ ± 0.20	0.23^a^ ± 0.07
2,3-butanediol meso	mgL^-1^	261.79^b^ ± 90.02	149.95^a^ ± 49.67	268.37^b^ ± 114.69	127.00^a^ ± 56.95
2,3-butanediol racemic	mgL^-1^	857.45^b^ ± 247.02	539.24^a^ ± 106.61	844.74^b^ ± 280.11	407.67^a^ ± 94.05
Ethanol	% v/v	11.89 ± 0.29	12.04 ± 0.35	11.98 ± 0.46	11.98 ± 0.41

Principal component analysis (PCA) was applied to the matrix of multivariate data comprising concentrations of the metabolic compounds. Residual sugars resulted lower than 2 gL^-1^ in all experimental fermented wines, with the exception for five Sangiovese wines obtained with the strains R2, R4, R16, R22, and R23 originating from Aglianico del Vulture and two Aglianico wines produced by the strains R2 and R53 (originating from Aglianico and from Sangiovese must, respectively). Therefore, these seven wine samples were not included in the PCA analysis.

**FIGURE 5:**
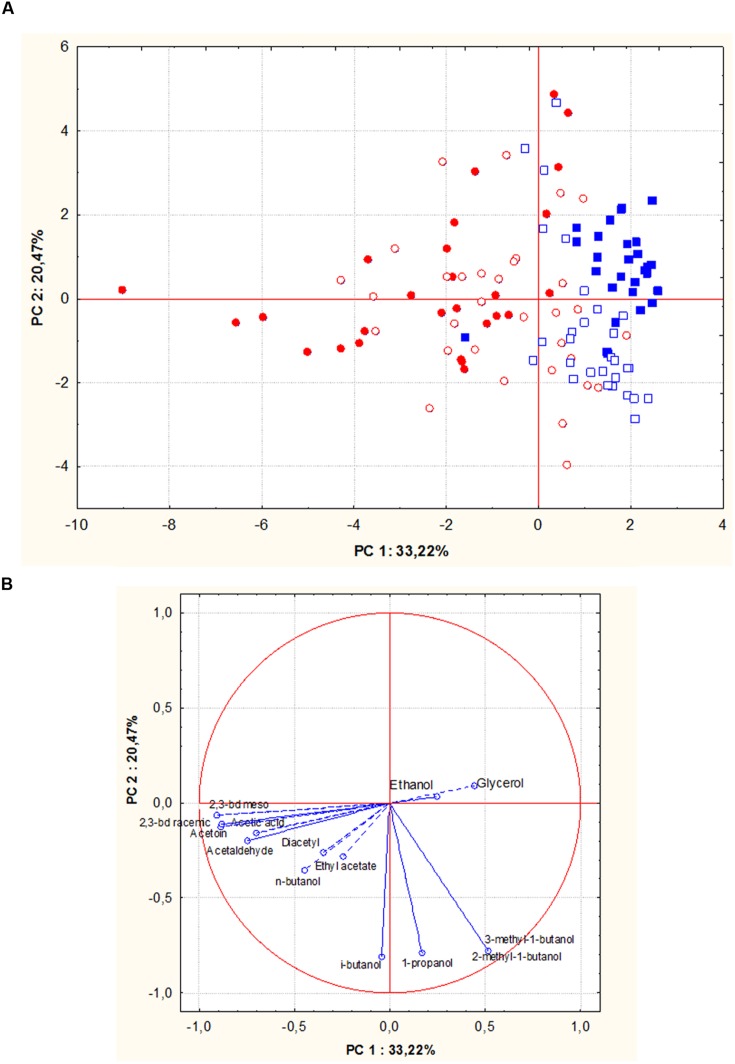
**Principal component analysis (PCA) plot based on by-products of alcoholic fermentations in Aglianico del Vulture and Sangiovese wine samples produced by *S. cerevisiae* isolates from Aglianico del Vulture and Sangiovese grape must.** (Blue square = wines obtained by isolates from Sangiovese: open symbols indicate Aglianico wines and close symbols indicate Sangiovese wines; red circle = wines obtained by isolates from Aglianico: open symbols indicate Aglianico wines and close symbols indicate Sangiovese wines); the scores **(A)** and variable loadings **(B)** for the two first principal components.

**Figures 5A,B** show PCA scores and loadings biplots, respectively, for all the experimental wines deriving from both grape musts fermentation by the 56 *S. cerevisiae* strains. Examination of the data by PCA showed that PC1 and PC2 accounted for 54% of variation in the dataset. Along the first component, independently of the fermented must, most of the wine samples grouped into two clusters according to the geographic origin of the fermenting yeast strains. In particular, 95% of wines produced by *S. cerevisiae* isolates from Sangiovese grouped in a cluster on the right of the plot, whereas 70% of wines obtained by *S. cerevisiae* isolates from Aglianico del Vulture were grouped into a more scattered cluster on the left of the plot. The first principal component correlated positively with 2-methyl-1-butanol, 3-methyl-1-butanol and glycerol and negatively with 2,3-butanediols.

**FIGURE 6:**
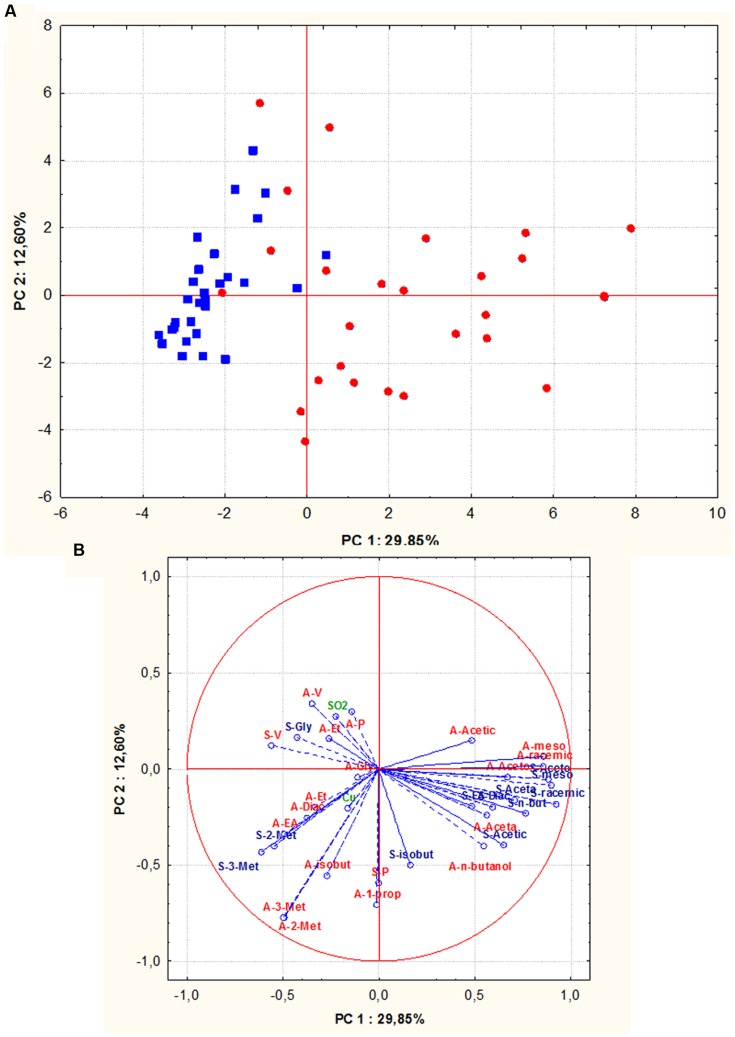
**Principal component analysis (PCA) of oenological properties (resistance to SO_2_ and copper, fermentative vigor and power, by-products of alcoholic fermentation) of *S. cerevisiae* isolates (red circle = isolates from Aglianico del Vulture, blue square = isolates from Sangiovese), the scores **(A)** and variable loadings **(B)** for the two first principal components.** Abbreviations for chemical measures in loadings are: red “A” refers to Aglianico del Vulture, blu “S” refers to Sangiovese, V, fermentative vigor; P, fermentative power; SO_2_, SO_2_ resistance; Cu, copper resistance; Et, ethanol; Acetic, acetic acid; meso, meso 2,3 butanediol; racemic, racemic 2,3 butanediol; Gly, glycerol; Aceta, acetaldehyde; Diac, diacetyl; Aceto, acetoin; EA, ethyl acetate; 3-met, 3- methyl 2 butanol; 2-met, 2- methyl 2 butanol; *n*-butanol, normal butanol; isobut, isobutanol; 1-prop, 1-propanol.

In addition, in order to evaluate whether *S. cerevisiae* isolates could group according to their geographic origin, all assayed oenological properties, including technological characters and by-products of alcoholic fermentations of both grape musts, were combined and analyzed by PCA. The biplot of the parameters considered, pointed out that along the first component all the Sangiovese isolates, except one, were positioned in the left quadrants while 88% of Aglianico isolates grouped on the right quadrants (**Figures 6A,B**). Therefore, a good relationship between *S. cerevisiae* isolates and their geographical origin was confirmed.

## Discussion

The concept of *terroir* for wine is classically considered as the result from the interaction between specific *Vitis vinifera* varieties and the local soils, geography, climate and agricultural practices (Van Leeuwen and Seguin, 2006). Recently, there is limited but increasing evidence showing that the microorganisms that influence vine growth, fermentation and wine style (as *S. cerevisiae* does) also exhibit regional differentiation (Lopandic et al., 2007; Gayevskiy and Goddard, 2012; Bokulich et al., 2014; Taylor et al., 2014; Knight and Goddard, 2015), supporting the concept that there could be a microbial aspect to *terroir*. In the present study, 63 *S. cerevisiae* isolates from two different grape musts (Aglianico del Vulture and Sangiovese) were characterized in order to assess the influence of geographic origin of these yeasts on their genetic and phenotypic patrimony. The results obtained by molecular fingerprinting using MSP-PCR with (GTG)_5_ and RFLP-mtDNA methods confirmed applicability and sensitivity of these methodologies for identification of different *S. cerevisiae* strains (Schuller et al., 2007; Orlić et al., 2010). Furthermore, these techniques were able to detect genetic differences between “Aglianico” and “Sangiovese” strains, resulting suitable methods to differentiate *S. cerevisiae* isolates based on their provenience as most of the isolates grouped according to their origin of isolation. The high genetic polymorphism found in the yeasts analyzed using the MSP-PCR and RFLP-mtDNA could be a result of a constant adaptation to the ecological conditions they are exposed to. Studies based on genetic and microbiological analyses suggest that in *S. cerevisiae* a significant part of the mechanisms affecting this genetic polymorphism occur during the vegetative phase of its growth cycle, where meiosis is a rare event (Puig et al., 2000; Perez-Ortin et al., 2002; Aa et al., 2006). That is to say, if yeasts reproduce clonally and they are constantly adapting to their particular environment, there must be a link between the genetic similarity of the strains and their geographic origin. It is well known that geographic or ecological isolation is one of the mechanisms involved in the process of speciation (Dobzhansky, 1951) as it creates a barrier for the genetic flux, so that strains coming from the same microenvironment will be more similar to each other than those from other geographic origin (Martínez et al., 2007). Our results, although based on the analysis of a small number of isolates, confirm that the wine production areas represent a reservoir of natural yeasts with peculiar genotypic profile, selected by the natural environment and by the interactions between yeasts and its environment (Guillamón et al., 1996; Martínez et al., 2007).

Further step of yeast characterization was addressed to identify potential relationships between strain origin and fermentation phenotype. Indeed, the yeast strain diversity might significantly affect the fermentation performance (Schuller et al., 2012; Tofalo et al., 2014). The data related to fermentative performance of the two groups of *S. cerevisiae* isolates, tested in the two isolation grape musts, seem to suggest that this parameter is influenced by both fermentation medium and source of yeast isolation. In fact, the isolates showed different fermentative performance in the two musts, with best results in Aglianico grape must, and Aglianico isolates exhibited higher fermentative performances than Sangiovese isolates (**Figures 4A–D**). As regards the fermentation substrate, the different yeast behavior could be correlated to the different composition of grape must. It has been underlined that wine fermentation conditions represent a combination of various stresses (osmotic, ethanol, acidic, nutrient limitation) that accentuate the metabolic differences between strains. Our results showed a certain influence of isolation origin (the grapes variety, in our case) on strain performance during the fermentation and might support the hypothesis that the autochthonous yeast strains are better adapted to the ecological and technological features of their own winegrowing area. These relationships between the origin and the phenotypes of some yeast strains could be due to physiological and metabolic adaptations in response to specific environmental conditions (Camarasa et al., 2011). Probably, “in the isolation grape must the strains are able to express their own better characteristics because they are better adapted to metabolize the precursors present in this grape must” (Capece et al., 2012). Indeed, in Sangiovese must five *S. cerevisiae* isolates from Aglianico were unable to complete the alcoholic fermentation. Furthermore, our data indicate significant correlations between the geographic relatedness of *S. cerevisiae* isolates and their effect on content of some compounds in the resulting wines in agreement with the results obtained by Knight et al. (2015). The PCA of experimental wines obtained by inoculating the *S. cerevisiae* isolates in Aglianico and Sangiovese grape musts (**Figure 5**) revealed that the samples were mainly grouped according to the geographic origin of the yeast strains. To our knowledge, few researches reporting the correlation between strain origin and fermentation phenotype are available until now. A study, aimed to analyze the variability of 36 *S. cerevisiae* strains, isolated from different grape varieties and from two very distant Italian zones (Mauriello et al., 2009), demonstrated that production of volatile aromatic compounds (VOC) allowed to differentiate the yeasts in function of isolation area. Indeed, *S. cerevisiae* isolated from Southern Italy grapes were able to produce more volatile compounds than those from Northern Italy. In a study performed on regionally genetically differentiated population of *S. cerevisiae* in New Zealand, Knight et al. (2015) demonstrated that these populations differentially affected wine phenotype. By evaluating the correlation between *S. cerevisiae* genetic distance and volatile chemical profile of wines, obtained by inoculating these strains, the authors found that these factors are correlated, confirming “the significant relationship existing between the genetic relatedness of natural *S. cerevisiae* sub-populations and their effect on resulting wine phenotypes” (Knight et al., 2015). However, these authors found that the chemicals responsible for the differences between regions are not consistently from any particular class. On the contrary, our results show that the production level of some compounds is correlated with yeast origin, independently from fermentation substrate. In particular, in both grape musts tested, *S. cerevisiae* from Aglianico produced significantly higher amounts of acetic acid, acetoin, acetaldehyde, *n*-butanol and 2,3-butanediols, while experimental wines produced by inoculating *S. cerevisiae* isolates from Sangiovese contained higher concentrations of 2-methyl-1-butanol and 3-methyl-1-butanol (**Table 1**). These results might be correlated to genotypic characteristics of yeasts. In fact, it is reported that the types and concentrations of metabolites produced by *S. cerevisiae* are significantly influenced by yeast genotype (Camarasa et al., 2011; Pretorius et al., 2012; Richter et al., 2013), while not exclusively genetically determined.

## Concluding Remarks

These findings support the need to unveil the indigenous *S. cerevisiae* population of specific areas and explore its natural biodiversity in order to produce valuable wines styles. The natural biodiversity of grape berries, grape juice, and winery environment, well correlated to each specific *terroir*, show a unique composition and represent great resources to winemaking. In fact, this work demonstrated that indigenous microorganisms are better adapted to the “chemical environment” of the grape must coming from a specific wine-producing area. The use of these strains as specific starter cultures can give distinct regional characteristics to wines. This suggests that safeguarding and exploiting natural biodiversity can allow the development of modern winemaking practices and the diversification of wine production, with tangible economic imperatives.

## Author Contributions

AC contributed to the design of the work, to the molecular characterization of yeasts, to the interpretation of data for the work, to draft the work and revising it; LG contributed to the design of the work, to the interpretation of data for the work, to draft the work and revising it; SG contributed to the molecular characterization of yeasts, to statistical analysis of data and to the interpretation of data for the work; SM contributed to chemical analysis of must and experimental wines, to statistical elaboration of data, RR contributed to the molecular characterization of yeasts, to the management of experimental fermentation, to the statistical elaboration of data; MV contributed to the design of the work, to the draft of the work and revising it, PR contributed to the design of the work, to the draft of the work and revising it, and ensured that that questions related to the accuracy or integrity of any part of the work were appropriately investigated and resolved.

## Conflict of Interest Statement

The authors declare that the research was conducted in the absence of any commercial or financial relationships that could be construed as a potential conflict of interest.
